# A pilot study demonstrating a non-invasive method for the measurement of protein turnover in skin disorders: application to psoriasis

**DOI:** 10.1186/2001-1326-2-12

**Published:** 2013-06-17

**Authors:** Claire L Emson, Sarah Fitzmaurice, Glen Lindwall, Kelvin W Li, Marc K Hellerstein, Howard I Maibach, Wilson Liao, Scott M Turner

**Affiliations:** 1 KineMed, Inc. 5980 Horton Street, Suite 470, Emeryville, CA 94608, USA; 2 Department of Dermatology, University of California at San Francisco, 1701 Divisadero Street, 3rd Floor, San Francisco, CA 94115, USA; 3 Department of Nutritional Sciences and Toxicology, University of California at Berkeley, 309 Morgan Hall, Berkeley, CA 94720, USA

**Keywords:** Psoriasis, Kinetics, Keratin, Skin, Stable isotopes

## Abstract

**Background:**

Previous studies of epidermal kinetics in psoriasis have relied on invasive biopsy procedures or the use of radioactive labels. We previously developed a non-invasive method for measuring keratin synthesis in human skin using deuterated water labeling, serial collection of tape strips and measurement of deuterium enrichment in protein by mass spectrometry. This powerful method can be applied to measure other skin proteins and lipids collected by tape stripping. Here, for the first time, we apply this technique to investigate the epidermal kinetics of psoriasis, the first step in defining a kinetic profile for normal skin versus activated or quiescent psoriatic skin.

**Methods:**

Psoriatic subjects were given ^2^H_2_O orally as twice-daily doses for 16–38 days. Affected and unaffected skin was sampled by tape stripping and washing (modified Pachtman method). Proteins were isolated from the tape strips by a method that enriches for keratin. Turnover times were determined by gas chromatography/mass spectrometry. Kinetic data were compared to transepidermal water loss (TEWL).

**Results:**

Deuterium-labeled protein from lesional psoriatic skin appeared at the skin surface within 3–8 days of label administration, whereas labeled protein from non-lesional skin requires 10–20 days to appear. Psoriatic skin had similar rate of growth despite varying anatomic location. Proteins recovered from tape strips were identified by nanoscale liquid chromatography/tandem mass spectrometry. Isolated peptides were >98% from keratin in uninvolved skin and >72% keratin in psoriatic skin. Revealing that one-quarter of all newly synthesized proteins in psoriatic skin are antimicrobial defense and other immune-related proteins. TEWL values were greater in lesional than non-lesional skin, suggesting barrier compromise in psoriatic skin despite increased clinical thickness.

**Conclusions:**

This simple, elegant, and non-invasive method for measuring epidermal protein synthesis, which can also be adapted to measure epidermal lipids, provides a metric that may reveal new insights into the mechanisms and dynamic processes underlying psoriasis and may also provide an objective scale for determining response to therapeutic agents in pre-clinical and clinical trials. This opens a pathway to the non-invasive study of kinetics of protein formation in psoriasis or other skin diseases.

## Background

Due to the hyper-proliferation and hyper-keratinization observed in psoriatic skin, there has been substantial work examining epidermal keratinocyte and keratin dynamics***.*** Abnormal epidermal turnover can impair skin barrier function and tissue repair; hence keratinocyte or keratin turnover may be critical in disorders of the skin as well as in response to therapy [1].

Dislipidemia is a further co-morbidity in this systemic disease. Skin lipid content is particularly critical for maintaining skin barrier function and regulating water loss. The stratum cornea is enriched with organized layers of intercellular lipids composed primarily of ceramides, cholesterol and fatty acids [2]. Psoriatic skin displays abnormal expression and abnormal location/distribution of lipids, and this appears to be at least partly related to disease severity. Therefore there may be significant changes in lipid kinetics in psoriatic individuals reflecting this defect in lipid homeostasis.

We have previously developed methods to measure the kinetics of keratin [3,4], triglycerides, fatty acids and cholesterol [5] using heavy water in human tissue and have also measured kinetics of complex lipids (e.g. galactocerebrosides) from brain myelin [6].

Protein synthesis can be conveniently measured by use of a continuous label administration, rise-to-plateau approach, based on the incorporation of deuterium (^2^H) from heavy water (^2^H_2_O) into nonessential amino acids (NEAA) in newly synthesized proteins [6]. This ^2^H_2_O technique has been used to measure the synthesis of proteins in muscle, bone, liver, lung and other tissues [6-10]. Similarly incorporation of ^2^H from ^2^H_2_O into cholesterol ester, free fatty acids or triglycerides can be used to determine the synthetic rates of lipid turnover.

When adhesive tape strips are applied to the skin surface in humans, a layer is removed composed primarily of lipids, keratin, and other epidermal constituents. The turnover rate of epidermal keratin from tape strips provides an accessible strategy for assessing psoriatic disease activity and treatment effectiveness. We have previously measured epidermal keratin synthesis by combining collection of tape strips with heavy water labeling and mass spectrometric analysis [4]. We observed that keratin and keratinocytes have equivalent rates of fractional synthesis in a psoriatic animal model and that keratin turnover from tape strips matches keratin turnover from tissue samples [4]. Here, we have applied our keratin synthesis method to measure skin protein turnover rates in involved and uninvolved skin of psoriatic individuals.

## Methods

### Study population

Four males (aged 49–67 years, mean 51.2 yrs, SD. 9.8) participated. All had dermatologist-confirmed severe plaque psoriasis. All subjects had no family history of disease and were concurrently receiving Goeckerman treatment at the University of California San Francisco (UCSF) Psoriasis and Skin Treatment Center. Goeckerman treatment involves exposing affected skin areas to ultraviolet B phototherapy and crude coal tar application daily, Monday through Friday. Subjects had been on a variety of prior therapies including UVB or UVA phototherapy, methotrexate, psoralen, cyclosporine, etanercept, adalimumab, efalizumab, infliximab, and alefacept but all were considered recalcitrant to treatment. None of the subjects had systemic treatment for psoriasis for at least one month or phototherapy for at least 2 weeks prior to study onset. Subjects were instructed to use nothing but emollients or moisturizers at the areas being sampled. Two plaques and two unaffected areas were sampled from each subject. Sampled areas were blocked from receiving Goeckerman treatment by daily application of Telfa pads (Covidien, Mansfield, MA) secured with micropore tape (3 M, St. Paul, MN). The study was approved by the Committee on Human Research at UCSF. All participants gave written informed consent. Declaration of Helsinki protocols were followed.

### Heavy water (^2^H_2_O)

Subjects drank 50 ml of 70% deuterated water (^2^H_2_O) twice daily Monday through Friday for 16 to 38 days. Saliva samples were collected on the day of tape stripping as previously described [4]. Salivettes were stored at −20°C until analysis by mass spectroscopy.

### Transepidermal water loss measurements and skin washing procedure

Transepidermal water loss (TEWL) was measured using an evaporimeter (Delfin VapoMeter™, Delfin Technologies Ltd., Finland). The probe was held perpendicular to the skin surface until a stable TEWL value was established (10–20 seconds). Two measurements were done at each of the 4 sites sampled. Skin washing was by a modified Pachtman method [11] in which a plastic cylinder 2.4 cm in diameter was pressed against the skin. One milliliter of wash solution (0.1% Triton X-100) was placed in the cylinder, and the skin surface was rubbed briskly with a Teflon policeman for 5 seconds. The solution containing scrubbed skin cells was collected with a sterile pipette, placed in Eppendorf tubes, and stored at 4°C until analysis. Details on measuring TEWL are summarized in [12].

### Tape stripping procedure

For each of two affected and two unaffected sampling sites, standard adhesive disks (D-Squame, CuDerm. Dallas Texas) with a 2.2 cm diameter were placed on the skin under a standard weight of 500 gm for 3 seconds then were removed from the skin using forceps. Twenty sequential D-Squame disks were taken from one area in each site. Tape strips were collected every 2–5 days, each time moving the collection area within each site. Sampling times were chosen to coincide with Goeckerman clinic visits and around subject availability to maximize compliance. Disks were measured by infrared densitometry [13], then were pasted onto laminated collection cards. Collection cards were stored at −20°C until analysis. A single physician conducted all tape strip collections. Additional methodological details are found in [14].

### Stratum corneum infrared densitometry

Twenty sequential tape strippings were placed adhesive side up on standard collection strip for a broad slit infrared densitometer (Squame Scan™ 850A Heiland electronic, Wetzlar, Germany). Stratum corneum per tape strip is indirectly determined by optical absorbance [15].

### Keratin preparation

Skin keratins were isolated from tape strips as previously described [4]. Briefly, a piece cut from each tape strip was placed in a microcentrifuge tube and washed in a high salt/mild detergent buffer to extract non-keratin proteins. The wash was discarded and keratins were solubilized in 1% SDS in water by heating at 100°C. Tape strips were removed from the tubes and the SDS-keratin complex was precipitated by adding KCl to the solution. Pellets were resuspended in 6 N HCl for hydrolysis to amino acids preparatory to derivitization and GC/MS analysis.

Pre-stripping skin washings were centrifuged 15 min at 18,000 x g in microcentrifuge tubes and separated into supernatant and pellet. The resulting pellets were processed as for tape strips, starting with the high salt/detergent washing. The supernatants were taken to 20% trichloroacetic acid, incubated overnight at 4°C, and centrifuged 15 min at 18,000 x g to precipitate any soluble proteins. These pellets were also processed as for tape strips.

### Peptide identification

Tape strip proteins were prepared for proteomic analysis using a protocol from the Vincent J. Coates Proteomics Laboratory at the University of California, detailed at their web site [16].

Pellets of the SDS-keratin complex precipitated by KCl as described above were mixed with Laemmli buffer (BioRad) and loaded on a 4-20% tris HCl gel (BioRad Criterion). The gel was run at 20 V until the dye front was about 1 cm into the gel and the largest of the prestained molecular weight markers (BioRad Precision Plus) were beginning to separate from the dye front. The gel was removed from the cassette, washed extensively in multiple changes of water and stained with Gelcode Blue® Coomassie stain (Pierce/Thermo Fisher). After additional extensive washing, gel lanes were broadly cut to cover the range of the molecular weight markers. Cut gel lanes were digested with trypsin and extracted per the University of California protocol [17] and submitted to that facility for analysis. Samples were run on a Thermo LTQ XL linear ion-trap mass spectrometer with LC-nanoelectrospray source. The program Sequest [18] identified peptides and proteins from the complete human database.

### Analysis of body ^2^H_2_O enrichments in body water

^2^H_2_O enrichment in saliva samples was measured as previously described [17,19]. Analysis of the deuterium content was performed by quadrupole GC/MS (Agilent 5973/6980) in negative chemical ionization mode (NCI-GC/MS), with helium as carrier and methane as reagent gas.

### Calculation of fractional synthesis

In order to determine the fractional synthesis of keratin, alanine from the acid hydrolysates was modified to pentofluorobenzyl derivatives and by GC/MS as previously described [7]. Briefly, the proteins were hydrolyzed by incubation in 6 N HCl for 16 hours, dried under vacuum and then suspended in a solution of 50% acetonitrile and 50 mM K_2_HPO_4_ pH 11. Twenty microliters of pentafluorobenzyl bromide were added before a 1 hour incubation. Derivatives were extracted into ethyl acetate. The derivatized alanine was analyzed by GC/MS, performed in the negative chemical ionization mode. Incorporation of ^2^H into protein-bound alanine was calculated as the molar fraction of molecules with one excess mass unit above the natural abundance fraction (EM1). Fractional turn over (f) was calculated as the ratio of the EM1 value in protein-bound alanine at the maximal value possible (representing 100% newly synthesized protein [7]) at the time-averaged body ^2^H_2_O enrichment present until the time the tape strip was obtained.

## Results

### Appearance of labeled protein in psoriatic lesions is rapid and the detected proteins are nearly completely newly synthesized material

**Figure 1:**
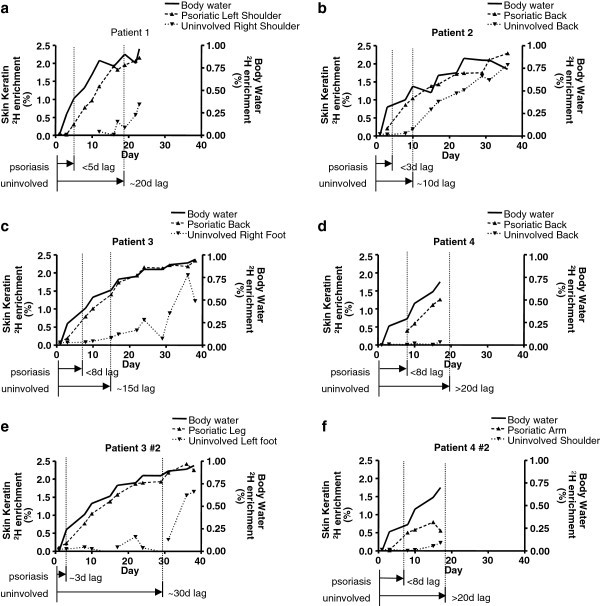
**Skin keratin enrichments.** Measured skin keratin enrichments for both psoriatic and uninvolved skin from four individuals **(a-d)**, based on deuterium incorporation in alanine, are plotted together with measured body water enrichments. Some individuals were assessed at two anatomical locations **(e-f)**. Deuterium incorporation at the surface of psoriatic skin lags body water levels by only a few days, whereas the lag is much longer in uninvolved skin. Body water enrichment has been demonstrated to be related to alanine enrichment by a factor of 2.7 and has been plotted here using that correlation.

Deuterated proteins were detected in tape strips from psoriatic lesions as early as 3 days after starting heavy water administration (Figure 1). Label was observed in all samples from lesions between 3–8 days after label administration. Because the first time point sampled was on day 3, initial appearance time may have been even sooner. Moreover, the protein isolated was nearly 100% newly synthesized, demonstrating that the entire stratum corneum compartment in psoriatic subjects is replaced in less than 5 days.

### Protein turnover in uninvolved skin is slower than that of involved psoriatic lesions

Skin tape strips collected from the same individuals but from uninvolved skin had significantly delayed appearance and slower turnover rates (Figure 1). Appearance of label was detected beginning around 10–20 days after the start of labeling. These results are consistent with our published data of keratin turnover rates determined from healthy, non-psoriatic individuals [4]. Although psoriasis is considered a systemic disease, these results indicate that the kinetics of uninvolved skin in psoriasis patients were comparable to normal skin and did not exhibit an increased basal turnover rate. Lag time for appearance of label did not correlate with subject age for involved or uninvolved skin.

### Different anatomical locations do not display dramatically different kinetic rates

**Figure 2:**
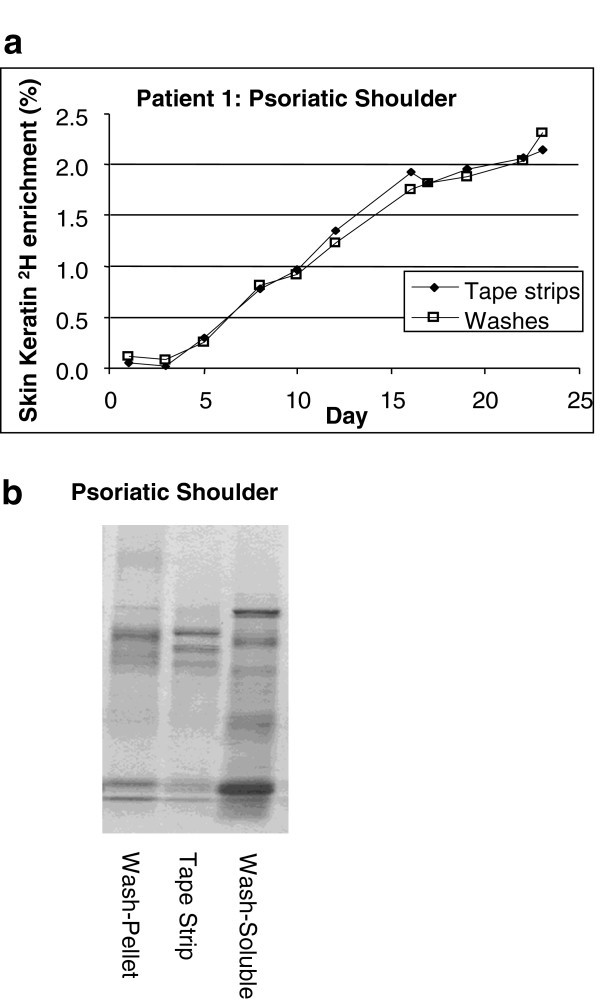
**Protein identification of soluble and insoluble skin proteins.** Prior to tape stripping, the skin surface was gently washed. The washes from psoriatic lesions contained abundant protein. Turnover rates of wash derived proteins were compared to tape strip proteins. The wash liquid was collected, centrifuged to separate soluble and insoluble fractions (tape strip processing removes soluble proteins), and processed using the same washing/SDS extraction process used for tape strips. **a)**. Incorporation of deuterium from ingested heavy water in tape strips and in proteins from the wash. **b)**. SDS PAGE stained with coomassie blue comparing the proteins recovered from a tape strip (middle lane) with the soluble and insoluble fractions from the skin surface wash of the same subject.

We sampled two anatomical locations on each psoriatic subject. Observations of protein turnover rates in involved and uninvolved skin were similar between the two different sites (Figure 1). Rates were similar within subjects and were location independent. In addition to this intra-subject similarity, inter-subject turnover rates were also remarkably consistent. Protein from tape strips and protein pelleted from skin washings (Pachtman technique) gave the same values for label incorporation (Figure 2). Washings from psoriatic lesions contained abundant protein but there was not always enough material in washings of uninvolved skin to be evaluated this way.

### The kinetically assessed tape strip proteins collected were predominantly keratin

This method was originally designed to enrich for and focus on keratins, washing away the readily soluble proteins before extracting the less-soluble keratins. Proteins from the involved psoriatic skin had a more complex banding pattern by SDS PAGE (data not shown). The samples recovered from tape strips from the uninvolved and psoriatic skin of subject 1 were therefore analyzed for protein content by nanoscale liquid chromatography-tandem mass spectrometry (LC/MS/MS). Table 1 lists the proteins observed in the uninvolved skin and the most prominent of the proteins from the psoriatic skin. 98% of the peptides identified from the uninvolved tape strips were derived from keratin, as determined by LC/MS/MS spectral counts, consistent with our previous findings [4]. Of the identified peptides from involved skin tape strips, 72% were keratins. The different makeup of the surface proteins in the involved skin reflected the disrupted profile of the plaque with detection of inflammatory and antimicrobial defense proteins (S100A8, Histone H4, and SERPINB4) as well as epithelial organizational proteins (Plakoglobin). A technical point worth noting regarding kinetics, however, is the near 100% fractional replacement of protein observed in epidermal skin strips from psoriatic subjects (Figure 1). This finding means that the keratin that is present is fully and rapidly turned over, regardless of the presence of contaminant proteins. Thus, the altered kinetics of label appearance in proteins from psoriatic patients does not represent labeling of inflammatory proteins but must primarily reflect turnover of keratin itself.

**Table 1:** Proteins observed by mass spectrometry from uninvolved and psoriatic skin of a single patient

**Uninvolved skin**	**# of unique peptides**	**total peptide count**	**mol wt (Da)**	**peptide count/mol wt x 10**^**3**^
Keratin 10	34	91	58827	1.55
Keratin 1	37	95	66039	1.44
Keratin 2	34	66	65433	1.01
Keratin 6A	10	24	60045	0.40
Keratin 77 (Keratin 1B)	16	24	61801	0.39
Keratin 5	11	20	62378	0.32
Keratin 14	5	7	51622	0.14
Glycine-N-acyltransferase isoform b	2	2	18506	0.11
Keratin 6 irs4	3	6	57865	0.10
Glyceraldehyde-3-phosphate dehydrogenase	2	3	36053	0.08
Keratin 9	4	5	62064	0.08
Keratin 5b	3	4	56866	0.07
Transglutaminase 3 precursor	2	2	76731	0.03
**Psoriatic skin**	**# of unique peptides**	**total peptide count**	**mol wt (Da)**	**peptide count/mol wt x 10**^**3**^
Keratin 6A	58	191	60045	3.18
Keratin 10	46	147	58827	2.50
Keratin 1	50	128	66039	1.94
Keratin 14	36	92	51622	1.78
Keratin 2	34	81	65433	1.24
Keratin 5	29	58	62378	0.93
Keratin 17	19	44	48106	0.91
Keratin 16	22	46	51268	0.90
Histone cluster 4	4	8	11367	0.70
S100 calcium-binding protein A8	3	7	10834	0.65
Serine (or cysteine) proteinase inhibitor, clade B, member 4	6	14	44854	0.31
Pyruvate kinase, muscle isoform 2	9	18	58062	0.31
Junction plakoglobin	12	22	81745	0.27

Proteins observed by peptide mass spectrometry from uninvolved and psoriatic skin of a single patient. For each protein the number of unique peptides and the total peptide count, which includes multiple observations of the same peptide, are listed. The peptide count has been divided by the molecular weight and proteins are listed in order of the resulting product. All 13 proteins identified from the uninvolved skin of this individual are listed together with the first 13 (of 66 total) proteins seen in psoriatic skin.

### TEWL data demonstrated decreased barrier integrity of involved psoriatic skin

**Figure 3:**
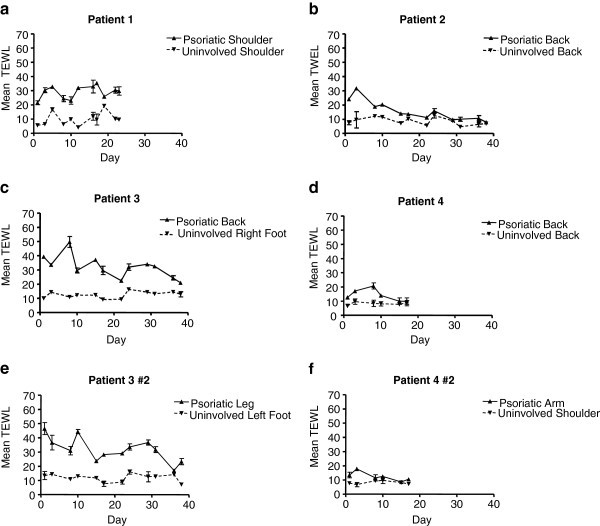
**Transepidermal water loss from psoriatic subjects.** Trans-epidermal water loss (TEWL) for both psoriatic and uninvolved skin from four individuals **(a-d)** was measured by contacting an evaporimeter to the skin surface. Some individuals were assessed at two anatomical locations **(e-f)**. TEWL measurements comparing psoriatic skin and non-lesional control skin is shown as a function of time. Each data point is an average of 2 values with standard deviations as indicated.

Transepidermal water loss (TEWL), a measure of epidermal water barrier function, was increased in the involved skin, demonstrating that the active psoriatic process is associated with decreased barrier function (Figure 3). An optical densitometry measure of tape protein levels was performed but yielded unreliable results due to the uneven adherence of psoriatic scale to the tape strips (data not shown).

## Discussion

There have been previous attempts to measure cell kinetics in both normal and psoriatic skin. Methods that have been used to measure the proliferative activity of the skin include bromodeoxyuridine (BrdU), DNA flow cytometry (FCM), cell cycle markers such as Ki-67 antigen and radio-isotopic tracers. Increased DNA synthesizing cells have been demonstrated in the psoriatic epidermis using methods, including tritiated thymidine and Ki-67 staining. In-vivo tritium labeling studies by Weinstein from the 1980’s predicted a cell cycle time of 311 hr in normal epidermis, 8 fold longer than the 36 hr cell cycle period in psoriatic epidermis [20].

Using the technique applied in the present study, our previous clinical studies measuring keratin synthesis in normal subjects demonstrated a transit time of about 18 days from the start of heavy water administration until label appearance at the skin surface [4]. A degree of variability in keratin synthesis kinetics was also observed among normal subjects. Here, we demonstrated similar appearance times for uninvolved skin from psoriatic individuals. This implies that basal turnover rates in skin are similar in psoriatic and non-psoriatic individuals, and that abnormal keratin kinetics are only observed in skin undergoing active disease. We also observed similar keratin turnover rates in uninvolved skin in different anatomic locations on the same individual, again suggesting consistent basal skin kinetics for these subjects except in areas of psoriatic plaques.

Most strikingly, labeled protein (predominantly keratin) was detected in lesional psoriatic skin within as little as three days following heavy water administration. Furthermore, as well as the rapid appearance time of labeled keratin on the skin surface, the keratin that was detected appeared to be nearly 100% newly synthesized during the labeling period. The calculated fractional synthesis rate (Figure 1) reflects the rate of protein synthesis but does not factor in the total protein present in the skin. Since keratin expression is elevated in psoriatic individuals, our observed increased fractional synthesis rate of keratin and rapid time to appearance indicate that the absolute keratin synthesis rate (mg new keratin per day) must be dramatically increased in psoriatic skin during active disease. Despite the small scale of this study, with limited subject number and sampling times, the difference in keratin turnover between involved and non-involved skin is striking and consistent.

All subjects had similar disease severity being diagnosed with severe plaque psoriasis (Table 2). Since subjects were deliberately selected to have severe plaque psoriasis we did not observe a correlation between synthesis rates and disease severity. Subject 3 had the most number of previous treatments including multiple systemic treatments and multiple biologic therapies. Despite his high severity, Subject 3 did not display the shortest or longest lag for involved or uninvolved skin (8 and 15 days respectively). We did not observe a correlation between demographics, disease severity or previous treatment with the keratin kinetics however with such a small number of subjects this study is not powered to reveal these differences.

**Table 2:** Subject demographics and clinical information

**Subject**	**1**	**2**	**3**	**4**
Age	49	49	41	67
Gender	M	M	M	M
BMI	25.7	32.5	32.1	31.4
Disease severity	Severe plaque psoriasis	Severe plaque psoriasis	Severe plaque psoriasis	Severe plaque psoriasis
Family history	No family history of psoriasis.	No family history of psoriasis.	No family history of psoriasis.	No family history of psoriasis.
Previous therapy	UVB phototherapy and methotrexate	UVB phototherapy	UVB and psoralen UVA phototherapy, methotrexate, cyclosporine, etanercept, adalimumab, efalizumab, infliximab, and alefacept	UVB and psoralen UVA phototherapy

Demographic and clinical data of the four subjects is shown.

Clinically the PASI score is considered the gold standard of disease activity. However the PASI score is not entirely objective and may be subject to significant inaccuracy (reviewed in [21]). Instead we utilized trans-epidermal water loss as a quantitative measure of stratum corneum dysfunction (Figure 3). This readout could distinguish between involved and uninvolved skin, highlighting the compromised barrier function of psoriatic skin and corroborating the kinetic data, but demonstrated a high degree of noise in day to day readings across the time course which would make it unsuitable for longitudinal assessment of response to therapy in a clinical trial. We also assessed subject disease activity using an optical readout. “SquamScan” (data not shown) did not reveal any significant difference between involved and uninvolved skin. Whilst diagnosis and management of individuals by the clinician is not hampered by the noise and insensitivity of these metrics it highlights the need for quantitiative tools for use in clinical trials to assess responses to novel therapies. Future studies could apply the keratin synthesis biomarker to cross sectional studies to measure the kinetic rates of variable disease severities or longitudinal studies examining kinetics in response to treatment to develop therapeutics and further the understanding of keratin dynamics in this disorder.

The non-invasive nature of the tape strip approach described here is simple and easily applied in a clinical setting. However, by collecting total skin proteins the differential composition of skin proteins between involved and non-involved skin must be considered. This method measures the weighted average of all protein in the sample and thus is biased to the most abundant. Accordingly, we performed LC/MS/MS to identify the proteins that were being kinetically characterized.

The proteins in Table 1 are ordered by dividing the peptide count, the total number of times peptides from a given protein are observed, by the molecular weight. This yields a number which is crudely related to abundance in the sample. Although there are some problems with using this ordering method (such as variations in efficiency of peptide ionization, variation in peptide resistance to proteolysis or poor peptide chromatograph) it has been demonstrated that abundance changes between related samples can be analyzed by this method [22].

Keratins 6A and 14 were greatly increased in apparent abundance relative to keratins 1 and 10 in psoriatic skin as compared to uninvolved skin. This data supports previous understanding of psoriatic lesions; keratins 6A and 14 are more prominently present in psoriatic than uninvolved tissue and keratins 16 and 17 are notably expressed in psoriatic tissue but are undetected in uninvolved skin [23].

Additional observations include that greater than 98% of all peptides observed in the uninvolved skin were from keratins. Thus, protein isolated from uninvolved skin by this method is almost all keratin [4]. Isolates from psoriatic skin are more complex; 66 proteins were detected. Keratins comprised only about 73% of all peptides observed in psoriatic skin, although the first 8 proteins were all keratins. The non-keratin proteins which appeared to be moderately abundant in this psoriatic tissue were predominantly proteins associated with epithelial organization (Plakoglobin), or epithelial innate immune responses such as inflammation (SERPINB4, S100A8) or antimicrobial (Histone H4). Many of these proteins have been previously demonstrated to be elevated in psoriatic lesions [24-27]. However, because essentially 100% of protein observed in epidermal skin strips from psoriatic subjects was newly synthesized (Figure 1), the altered kinetics of label appearance in proteins from psoriatic patients cannot simply represent labeling of inflammatory proteins but primarily reflects turnover of the predominant protein, keratin.

Another point worth noting is that our approach yields an average turnover rate for the extracted proteins. This approach works well for healthy epidermis which is thought to be composed largely of layers which work their way to the surface at a fairly uniform rate. Psoriatic skin is less ordered and could conceivably vary in turnover rates of some components. In the future, use of advanced dynamic proteomic techniques to pull out turnover rates of individual proteins from the labeling of peptides may be applied with the same heavy water labeling/skin strip collection methodology that is described here.

The current technique has distinct advantages over the traditional methods of determining cell proliferation that have been described previously. Many kinetic studies involve the labeling of human subjects or ex-vivo skin biopsies with radioactive tracers. Both BrdU and 3HdT are toxic and mutagenic, providing ethical and practical considerations preventing their use. Stable isotopes such as deuterium (^2^H) have a long history as safe, effective methods for measuring synthesis of molecules in experimental animals and humans. Because deuterium is safe for human use, it is easily translated into clinical studies. By using stable isotopes one can label for longer periods allowing the disease to be tracked over time, for example during a treatment or intervention. Further, many historical studies use punch biopsies; this is invasive and not suitable for already damaged lesional skin, especially since injury alone may induce further exacerbations in uninvolved skin (Koebner effect). Tape stripping is a less invasive method of sampling the skin surface and also allows us to take multiple samples from the same subjects over time. No Koebner effect was observed in any subject during this study. The safety and simplicity of this tape-stripping technique enables the possibility for much larger scale clinical studies, giving a more accurate determination of kinetics based on a larger population size.

Administration of deuterium oxide and collection of skin samples is very easy making this assay highly attractive; however as with any new assay feasibility and cost must be considered. Unlike other methods of assessing protein turnover which historically have used short term i.v. infusion of radiolabelled tracer or invasive punch sampling (discussed above) in this study subjects drink pre-bottled D2O at home and are then tape stripped. In our study tape strips were collected by a single person to minimize any sampling variability between subjects in such a small cohort and because the subjects were already visiting the clinic for pre-existing visits. However skin collection could also be carried out by the subject in an outpatient setting as tape strip collection is painless and requires minimal training. Following collection tape strips are stuck onto a laminated card and can be stored in the subjects’ freezer until collection. It is conceivable to imagine a kit mailed to the subjects home of D2O, tape strips and collection cards. The abundance of keratin in the tape strip sample means protein isolation is relatively easy for any chemical laboratory. We envision this method being most applicable to interpretation of clinical trials rather than routine disease management of patients. In this scenario access to a mass spectrometry instrument and chemical lab is not limiting. Additionally, new mass spec methods such as MRM or SISCAPA, are high through put and have been used to translate biomarker approaches from discovery to clinical trials. These methods can be used to measure a targeted protein of interest from a complex biological sample and this method could be adapted to this platform.

The availability of a rapid, quantitative biomarker of disease and treatment efficacy, either protein or lipid, would have wide spread clinical applications. It would enable the basic study of disease pathogenesis and allow for the rapid assessment of novel therapeutics (decreasing the time and cost of clinical trials and reducing patient exposure to new agents with unknown side effects). We predict that our keratin synthesis biomarker would be especially suitable to detecting early response to treatment prior to detection by traditional clinical metrics. An objective marker of psoriatic disease activity, such as the keratin kinetic biomarker described here, could also be used to assess the severity of other psoriasis sub-types such as palmoplantar psoriasis, guttate psoriasis, inverse psoriasis, and erythrodermic psoriasis. These subtypes are not well measured by the current scales which were designed to evaluate plaque psoriasis. The method described here is potentially applicable to all phenotypes of psoriasis.

As well as analyzing keratin turnover as described above, we can obtain other skin constituents from the tape strip. We have previously developed methods to measure the kinetics of lipids [5,6] and cell proliferation [28,29] using heavy water in human tissue. The adaption of this tape strip technique to enable kinetic assessment of specific skin cells, lipids or proteins may yield further insights into the mechanisms behind this complex disorder. We have recently devised methods of measuring multiple protein kinetics simultaneously [30-32] which could further expand the utility of this method in looking at the pathology of psoriasis or response to treatment.

## Conclusion

In summary, we have demonstrated that keratin turnover is dramatically accelerated in psoriatic lesions while uninvolved psoriatic skin has keratin turnover rates similar to non-psoriatic individuals. Although only a pilot study this suggests that keratin synthesis kinetics may be a sensitive biomarker of psoriasis which could be used to assess individual disease activity or to provide a quantitative measure of response to treatment in clinical trials. This kinetic method of assessing skin activity or skin turnover is non-invasive and safe. Application of this technology could be used as a quantitative means to standardize either basic science or clinical studies of psoriasis, or of other skin conditions such as icthyosis or aging skin.

## Abbreviations

H: Deuterium; H2O: Deuterated water; EM1: One excess mass unit above the natural abundance; F: Fractional turn over; GC/MS: Gas Chromatorgraphy Mass Spectrometry; LC/MS/MS: Nanoscale liquid chromatography-tandem mass spectrometry; NEAA: Non-essential amino acids; TEWL: Transepidermal water loss.

## Competing interests

C. Emson, G. Lindwall, K. Li and S. Turner are all employees and have stock options in Kinemed. M. Hellerstein is a consultant and stock holder at KineMed.

## Authors’ contributions

CE was responsible for study design, clinical documentation, heavy water production and distribution, data analysis, data interpretation and drafting the manuscript. SF was responsible for clinical sample collection, TEWL analysis and manuscript review. GL was responsible for sample preparation for mass-spec, data analysis and interpretation and manuscript review. KL was responsible for data interpretation and manuscript review. MH was responsible for data interpretation and manuscript review. WL was responsible for subject recruitment, clinical evaluation and manuscript review. MH was responsible for study design, data interpretation and manuscript review. ST was responsible for study design, data interpretation and manuscript review. All authors read and approved the final manuscript.
